# A Rapid Virus‐Free Method for Producing Influenza HA Immunogen Needed for Preparation of Influenza Vaccine Potency Antisera Reagents

**DOI:** 10.1111/irv.70024

**Published:** 2024-10-23

**Authors:** Marcus Odin, Falko Schmeisser, Jackeline Soto, Jerry P. Weir

**Affiliations:** ^1^ Division of Viral Products, Laboratory of DNA Viruses, Center for Biologics Evaluation and Research Food and Drug Administration Silver Spring Maryland USA

**Keywords:** influenza vaccines, single‐radial immunodiffusion assay, vaccine potency

## Abstract

**Background:**

The potency of inactivated and recombinant influenza vaccines is measured using the single‐radial immunodiffusion (SRID) assay. The strain‐specific antigen and antibody potency reagents required for the assay are prepared and distributed by regulatory agencies to ensure vaccine standardization, but timely reagent production is always challenging. This poses unique concerns for rapid pandemic responses. Alternative methods have been described for generating strain‐specific potency antibody reagents without the need for live influenza virus, but such methods are infrequently used, suggesting the need for additional antigen expression approaches.

**Methods:**

We describe a rapid process using a mammalian expression system to produce recombinant influenza hemagglutinin (rHA). This platform was used to generate rHA from two H5 clade 2.3.4.4 influenza viruses, in both soluble ectodomain or full‐length HA forms, and a soluble ectodomain rHA from an influenza H2 virus.

**Results:**

The purified rHAs were used as immunogens to produce HA antibody reagents that were tested for suitability in the SRID assay to accurately measure the potency of inactivated pandemic influenza vaccines. Antibody reagents generated to either ectodomain or full‐length rHA worked well in the SRID assay and resulted in vaccine potency values equivalent to those generated with standard reference antibodies.

**Conclusions:**

The results demonstrate that rHA produced from a simple mammalian cell transfection method can be used to generate HA antibody suitable for use in the influenza vaccine SRID potency assay and suggest a practical means by which an extensive library of pandemic reagents can easily be prepared in advance of and during an influenza emergency.

## 1 Introduction

The potency of inactivated and recombinant influenza vaccines is measured using the single‐radial immunodiffusion (SRID) assay [1]. The assay measures the content of virus hemagglutinin (HA) in the vaccine by comparison to the assigned HA value of a homologous HA Reference Antigen using a strain‐specific influenza antibody. The Reference Antigen used in the SRID assay is typically a preparation of whole virus, inactivated, aliquoted, and lyophilized, whose HA content has been determined by the World Health Organization Essential Regulatory Laboratories (ERL) through a collaborative exercise [2]. The corresponding strain‐specific antibody used with the Reference Antigen in the SRID assay is usually prepared by immunization of sheep using soluble HA that has been removed from the virus particle by bromelain treatment. The antigen and antibody potency reagents for the assay are prepared and distributed by regulatory agencies for use by regulators and vaccine manufacturers and ensure standardization of vaccines made by different manufacturers. Timely reagent production is challenging as reagents are strain‐specific and must be produced for each new virus strain in a vaccine. Thus, this process always poses a potential bottleneck in influenza vaccine production and is a concern for a timely pandemic response.

An alternative approach for generating strain‐specific potency antibody for use in the SRID assay has previously been described [3]. The strategy involved construction of DNA and modified vaccinia virus Ankara (MVA) vectors that expressed HA. The results demonstrated that HA‐expressing vectors can be generated in the absence of influenza virus availability and without reliance on the success of a bromelain treatment purification step, providing an alternative method for the production of the SRID potency antibody reagents. A few years later, when inactivated influenza vaccines were developed by manufacturers in response to the emergence of a novel H7N9 virus in 2013 [4, 5], difficulties were encountered in preparation of the antibody potency reagent using traditional approaches. By applying the alternative method previously developed to generate HA for immunization, suitable antibody reagents for these H7N9 inactivated vaccines were produced [6]. This antibody reagent was suitable for use in vaccine potency SRID assays and represented the first production of a potency antibody reagent prepared by an alternative method that was made available by a WHO ERL for general use by vaccine manufacturers and other regulatory agencies.

Despite the demonstrated feasibility of using alternative methods to prepare potency antibody reagents for pandemic influenza vaccines, and the obvious advantage of being able to start reagent work before virus is available, alternative methods for reagent preparation are infrequently used. One reason may be that the alternative methods previously described are not readily available for most laboratories, which suggests that additional antigen expression approaches are needed that can be easily transferred and rapidly implemented in regulatory laboratories.

We describe here a fast, simple process to produce the immunogen needed for immunization to prepare potency antibody for pandemic influenza vaccines using a mammalian cell culture transfection system. This platform was used to produce rHA from two H5 clade 2.3.4.4 influenza viruses, in both soluble ectodomain or full‐length HA forms, and a soluble ectodomain rHA from an influenza H2 virus. These rHA antigens were used as immunogens to produce HA antibody reagents that were suitable for use in the SRID assay to measure the potency of inactivated pandemic influenza vaccines.

## 2 Results

### 2.1 Expression and Purification of H5 Influenza rHA

**FIGURE 1:**
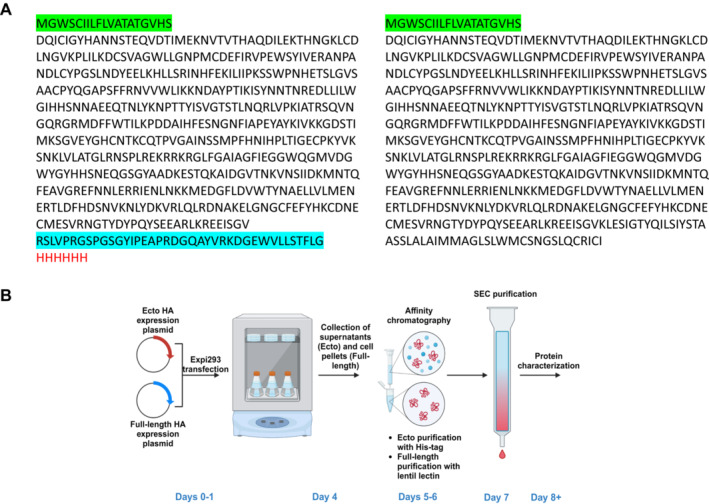
Cloning and purification strategy for influenza rHA expression. (A) Amino acid sequence of recombinant ectodomain A/Astrakhan/3212/2020 HA (left) and full‐length HA (right). The leader sequence is shown in green, the trimerization sequence in blue, and the His‐tag sequence in red. (B) Schematic diagram of the purification scheme used for Expi293 expressed recombinant ectodomain and full‐length influenza HA (created with BioRender.com).

Highly pathogenic avian influenza (HPAI) H5 viruses re‐entered North America, and subsequently the United States, at the end of 2021 and early 2022, evolving rapidly [7] and causing large outbreaks in both wild aquatic birds, commercial poultry [8], and dairy cows [9]; sporadic human infections have also been reported [10]. Subsequent genetic analysis indicated that these viruses were from the H5N1 clade 2.3.4.4b and that the hemagglutinin was closely related antigenically to the HA of a recent human H5N8 isolate A/Astrakhan/3212/2020 (A/Astrakhan) [11]. The HAs from these clade 2.3.4.4b viruses were also antigenically related to the HA of an H5N8 virus vaccine, derived from the clade 2.3.4.4c virus A/gyrfalcon/Washington/41088–6/2014 (A/gyrfalcon/Washington), that had recently completed preliminary clinical trials [12, 13]. To support vaccine reagent development for such influenza viruses with pandemic potential, we developed expression and purification methodologies for recombinant H5 HA that could be used for rapid production of an immunogen in the absence of live virus. In one approach, we constructed mammalian expression vectors expressing the hemagglutinin ectodomain of the A/gyrfalcon/Washington and A/Astrakhan H5 clade 2.3.4.4 viruses noted above. The expression strategy is shown in Figure 1A (left) for the A/Astrakhan sequence. The authentic HA leader sequence was replaced with a synthetic leader sequence as shown in green, and in addition, the transmembrane and cytoplasmic portions of the HA were removed and replaced by a synthetic trimerization sequence (blue) and a His‐tag (red) [14]. In a second approach, we constructed mammalian expression vectors expressing the full‐length HA of these two H5 HAs, with the leader sequence replaced as for the ectodomain constructs (Figure 1A, right).

The transfection and purification strategy is shown in Figure 1B. Expression plasmids were transfected into Expi293F suspension cultures and supernatants and cell pellets harvested 4 days later. Most of the full‐length HA remained in the cell pellet at this point but could be extracted with detergent treatment and purified by lentil‐lectin affinity chromatography and size exclusion chromatography (SEC). The ectodomain HA was effectively secreted into the supernatant and was purified by His‐tag affinity chromatography and SEC. The entire expression and purification procedure for either recombinant ectodomain or full‐length H5 HA could be completed in 8 days.

### 2.2 Characterization of H5 Influenza rHA Produced by Transfection of Expi293 Cells

**FIGURE 2:**
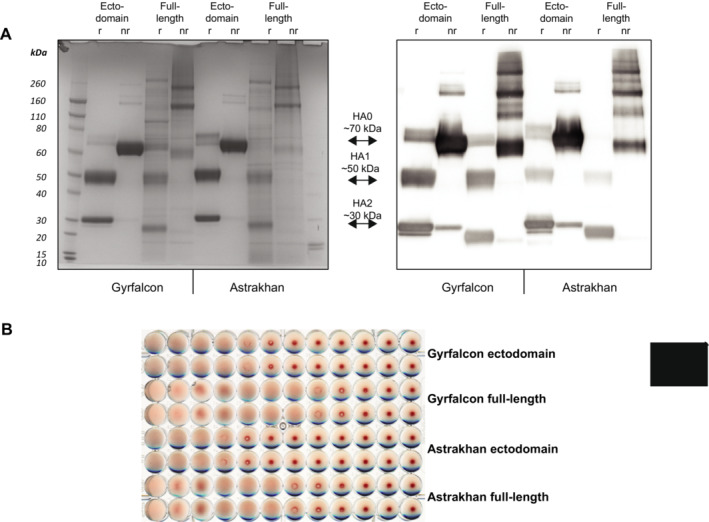
Characterization of Expi293 expressed H5 rHA. (A) Recombinant A/gyrfalcon/Washington and A/Astrakhan H5 HA from transfected Expi293 cells was purified and characterized by SDS‐PAGE under reducing (r) and non‐reducing (nr) conditions. Gels were stained with Coomassie blue (left) or transferred to nitrocellulose membranes for analysis by Western blot (right). (B) Purified rHA was analyzed for HA activity using chicken red blood cells and a starting protein concentration of 0.05 mg/mL.

The soluble H5 ectodomain rHA purified by His‐tag affinity chromatography from transfected Expi293 cells typically eluted as a single peak upon size exclusion chromatography (Figure S1). The purity of the ectodomain protein was demonstrated to be of high quality by SDS‐PAGE and Western blot analysis (Figure 2A) and hemagglutination of red blood cells (Figure 2B), indicated that the confirmation of the HA trimer conformation was conserved. The full‐length H5 rHA, without an added peptide tag, was gently extracted using the detergent *n*‐dodecyl‐β‐d‐maltopyranoside (DDM, 1%), and then enriched by lentil lectin affinity chromatography. The detergent concentration was gradually decreased during the washing and elution steps to a final concentration of 0.02% DDM, which was maintained during and following SEC. Protein eluted over a broad range during SEC, but fractions were individually tested for HA activity, and the fractions with the highest levels of HA activity were pooled for analysis (fractions 25–32, Figure S1). Although not as pure as the ectodomain rHA, the final full‐length rHA was greatly enriched, as shown in SDS‐PAGE and Western blot analysis (Figure 2A) and also hemagglutinated red blood cells (Figure 2B). Ectodomain rHA was typically greater than 95% pure, and full‐length rHA was approximately 70% pure. When ectodomain and full‐length rHA preparations were adjusted to the same amount of total protein, a higher level of HA activity was always measured for the full‐length HA.

### 2.3 Antibody Production to H5 rHA and Evaluation in the SRID Assay

**FIGURE 3:**
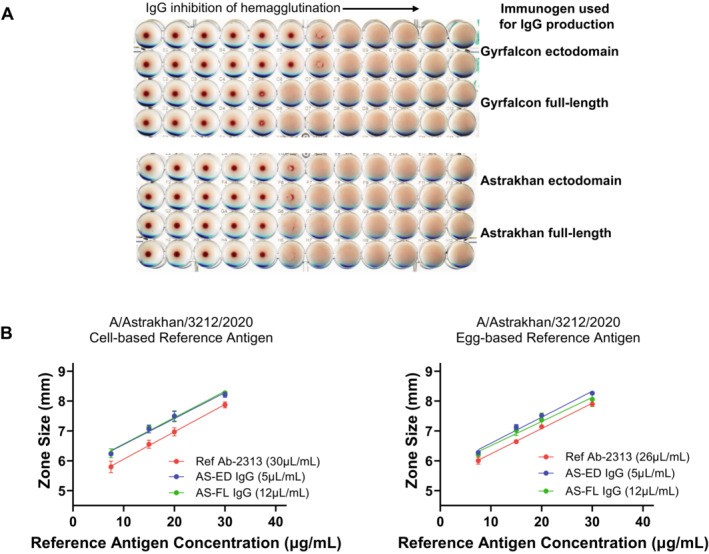
Characterization of H5 IgG prepared from rHA. (A) IgG preparations using the indicated rHA as an immunogen were analyzed for HA inhibition of the respective Reference Antigen, A/gyrfalcon/Washington Antigen H5‐Ag‐1514 (top) or A/Astrakhan Reference Antigen H5‐Ag‐2301 (bottom). The starting IgG concentration in the assay was 0.5 mg/mL. (B) Optimization of IgG concentration in the SRID assay. Dilutions of A/Astrakhan cell‐based (left) or egg‐based (right) reference antigens were analyzed by SRID using the A/Astrakhan reference antibody or the IgG antibodies made to the A/Astrakhan ectodomain (AS‐ED) or full‐length (AS‐FL) rHA. The zone size (mm) refers to the diameter of the measured precipitin rings.

Ectodomain and full‐length versions of A/gyrfalcon/Washington and A/Astrakhan rHA were used to immunize rabbits to produce polyclonal antisera to HA. IgG was purified from each immunization project for evaluation as potential SRID potency reagents. Each H5 IgG inhibited the HA activity of both A/gyrfalcon/Washington and A/Astrakhan reference antigens and there was no apparent difference in the inhibition activity among the four IgG preparations (Figure 3A).

Each recombinant HA‐generated polyclonal IgG was evaluated for suitability as an antibody reagent in the SRID potency assay in comparison to the designated reference antiserum for each. All of the IgG reagents produced clear easily readable precipitin rings in the SRID assay, suggesting their suitability for assay use, and a working concentration of each was determined using both egg‐based and cell‐based A/gyrfalcon/Washington and A/Astrakhan reference antigens (Figure 3B).

**FIGURE 4:**
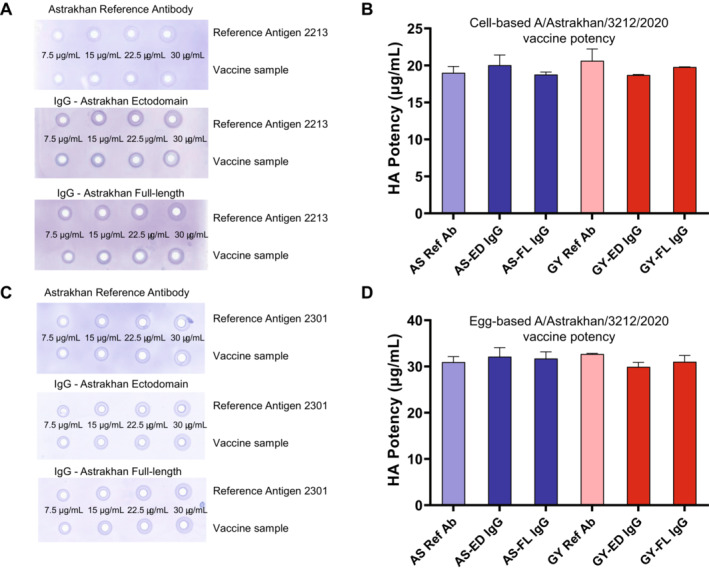
Potency determination of H5 vaccine samples. Representative SRID gels are shown for analysis of cell‐based A/Astrakhan (A) and egg‐based (C) vaccine. Potency of the cell‐based A/Astrakhan (B) and egg‐based (D) vaccines was determined using six antibody preparations. Potency values determined using homologous antibodies are shown in blue; potency values determined using heterologous A/gyrfalcon/Washington antibodies are shown in red.

Three pandemic influenza vaccines have been licensed in the United States, and each manufacturer uses a manufacturing process similar to that used for their seasonal influenza vaccine. In the interests of pandemic preparedness, the US Department of Health and Human Services (HHS) periodically contracts with these manufacturers of licensed vaccines to produce pilot lots of vaccine for clinical evaluation and two such pilot lots of vaccine were prepared using a recently developed A/Astrakhan/3212/2020 candidate vaccine virus (CVV) (www.clinicaltrials.gov—NCT05975840, NCT05874713). We obtained samples of both cell‐ and egg‐based A/Astrakhan vaccines, and tested their potency using the standard A/Astrakhan reference antibody and the four H5 IgG reagents that were prepared using rHA. Examples of SRID gels used in the analysis are shown in Figure 4A,C for the cell‐based vaccine and egg‐based vaccines, respectively. There was no significant difference in the potency values determined for the cell‐based A/Astrakhan vaccine sample regardless of whether the standard reference antibody prepared in sheep or the antibodies made to A/Astrakhan ectodomain or full‐length rHA made in rabbits were used in the assay (Figure 4B, blue bars). Similarly, potency values determined for the egg‐based vaccines were equivalent using either the standard reference antibody or the alternative A/Astrakhan antibodies (Figure 4D). Interestingly, the potency values obtained using the heterologous antibodies made to A/gyrfalcon rHA were also equivalent, regardless of antibody reagent used in the assay (Figure 4B,D, red bars). Taken together, these results indicated that rHA, either ectodomain or full‐length HA, produced in the Expi293 transfection system was suitable for preparation of an SRID potency antibody reagent for influenza vaccine evaluation.

### 2.4 Expression and Purification of an H2 rHA Ectodomain and Preparation of a Potency Assay Antibody Reagent

Having developed a fast method for influenza rHA production with H5, we wanted to expand our investigation to prepare potency reagents for other influenza viruses with pandemic potential. Although H2 influenza viruses have not circulated in humans as this subtype was replaced by H3N2 in 1968, H2 viruses similar to the H2N2 pandemic virus are still circulating in avian species with occasional spillover into swine [15] and remain a pandemic threat [16]. Accordingly, HHS recently decided recently to contract for production of a pilot lot of vaccine for the H2 virus A/chicken/Ohio/494832/2007 (A/chicken/Ohio) [17] for clinical evaluation (clinical trial identifier NCT05875961). As a result of that decision and the success of our rHA purification process, we generated and purified an H2 ectodomain for this virus HA using the same procedure described for the H5 ectodomains. Interestingly, expression of H2 produced almost exclusively HA0 rather than the HA0, HA1 and HA2 species observed when H5 was expressed in Expi293 cells (Figure S2). Presumably, this difference is due to the presence of the multi‐basic sequence in the H5 constructions at the HA1 HA2 junction. Nevertheless, as the H2 rHA was very pure (> 95%) and hemagglutinated red blood cells, we initiated an immunization project to produce an antibody reagent for this novel HA antigen. As the H5 antibody preparations did not indicate a difference between ectodomain and full‐length immunogens, we only prepared an ectodomain antigen for the H2 A/chicken/Ohio antibody investigation.

**FIGURE 5:**
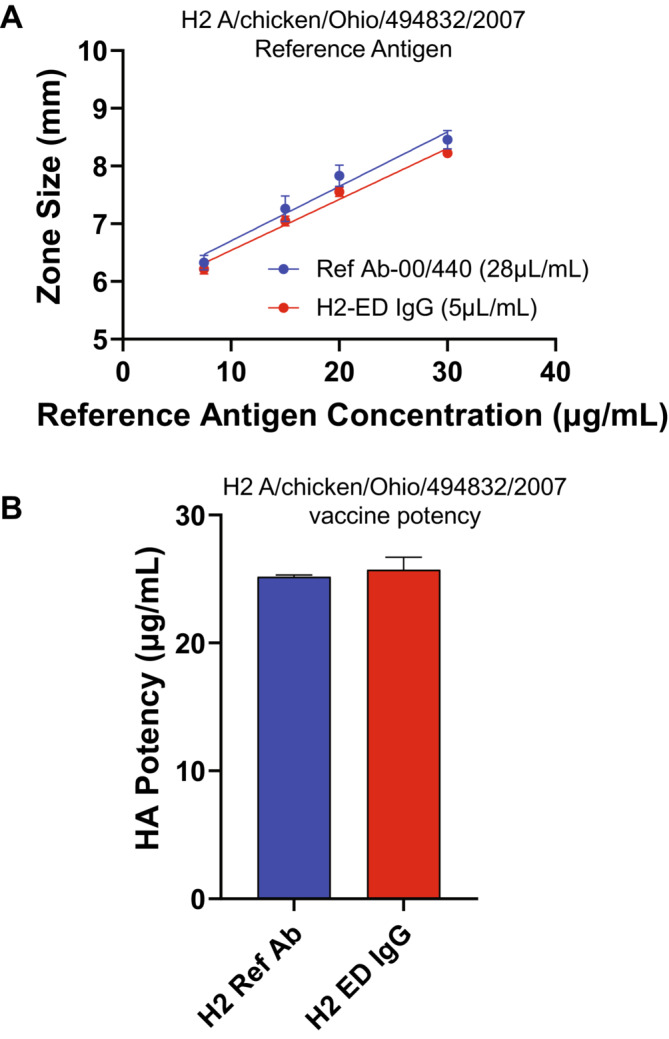
H2 IgG Characterization and Potency of H2 Vaccine. (A) Dilutions of H2 A/chicken/Ohio reference antigen were analyzed by SRID using the H2 reference antibody or the IgG antibodies made to the A/chicken/Ohio ectodomain (H2‐ED) rHA. The zone size (mm) refers to the diameter of the measured precipitin rings. (B) Potency of the H2 A/chicken/Ohio vaccine was determined using the H2 reference antibody or the IgG antibodies made to the A/chicken/Ohio ectodomain (H2‐ED) rHA.

Similar to the results with the H5 antibody studies, the H2 IgG produced clear easily readable precipitin rings in the SRID assay over the standard range of the assay (Figure 5A) and yielded a potency value for an H2 A/chicken/Ohio/494832/2007 vaccine sample that was equivalent to that obtained using the H2 reference antibody (Figure 5B).

## 3 Discussion

The primary goal of the studies described here was to develop a simple and fast process for producing the immunogen needed for immunization to prepare influenza vaccine potency antisera reagents without the need for live virus. The focus was on pandemic influenza virus reagents because speed and alternatives would be most critical during the emergence of a pandemic, and also because availability of live pandemic virus for reagent preparation might be problematic during early stages of a pandemic. While alternative methods that use rHA for producing influenza potency reagents have been described, and in some cases utilized, such methods are not widely employed. Typically, HA is prepared from either whole wild‐type or CVV virus particles by bromelain treatment, followed by partial purification of the soluble portion of the HA. While this process is fairly reliable, particularly for seasonal strains of influenza, problems are occasionally encountered such as the previously noted difficulty in preparing H7 potency antisera [6]. Such issues, in addition to the requirement for live virus, argue for the development of antigen expression approaches that can easily be implemented.

In the alternative process described here, an initial plasmid cloning and construction step, which can usually be completed within a couple of weeks, is followed by a transfection and HA antigen purification scheme that is fast, simple, and results in rHA preparations devoid of any other influenza proteins. The purity and yields are more than sufficient for immunization purposes and produce rHA that maintains functionality for hemagglutination. Typical final yields after affinity and size exclusion chromatography steps are in the range of 0.4 mgs (full‐length) to greater than 1 mg (ectodomain) per 100 mL of transfected Expi293F cells. Ectodomain rHA is regularly greater than 95% pure, whereas full‐length rHA is around 70%. Interestingly, the full‐length rHA consistently exhibited higher levels of HA activity compared with an equivalent amount of ectodomain rHA, suggesting the possibility that the trimeric ectodomain form of rHA might be somewhat less stable than the full‐length form or that the full‐length rHA might assemble into higher order structures than the ectodomain rHA. The full‐length rHA needed the inclusion of a low concentration of detergent to avoid precipitation; however, we did not investigate whether such a detergent component would have been beneficial for the stability of the ectodomain rHA.

Both ectodomain and full‐length forms of HA were capable immunogens for generating high titer polyclonal antibody that could be used in the influenza SRID potency assay. There did not appear to be any significant differences in the final antibody preparations as far as functionality in the SRID assay, and antibodies prepared against both ectodomain and full‐length rHA worked well in the SRID assay to determine the potency of actual vaccine samples, manufactured in either egg or cell substrates. There was no difference between the potency values of the vaccines determined using the rHA produced antibodies and the Reference Antiserum produced by traditional methods. Further, the antibody preparations made to the heterologous H5 HA from A/gyrfalcon/Washington also appeared to work well in the SRID assay of A/Astrakhan vaccines, suggesting that a perfect match of rHA may not be critical for production and use of an SRID antibody reagent. It is noted that A/gyrfalcon/Washington and A/Astrakhan HAs are genetically and antigenically similar [18], both being from H5 clade 2.3.4.4 viruses and differing by only 16 amino acids in their ectodomain. Further work will be necessary to determine whether cross‐reactive antibodies functional in the SRID can be predicted from genetic and antigenic data and if so, how closely related or similar the HAs used to generate the antibodies must be. Nevertheless, such information would be extremely valuable for pandemic readiness.

Because our goal was to compare several different rHA preparations, we prepared antibodies in rabbits rather than sheep, which is common for routine seasonal reagent preparation in order to obtain the quantities of antibody reagents needed to support seasonal vaccine production. However, there is nothing unique about sheep antibodies, as opposed to antibodies from other animal species, as far as functionality in the SRID assay. The use of in‐bred animals such as rabbits likely contributes to a reproducible antibody response that allows pooling of sera. In addition, although the traditional potency reference sera is used without further purification of IgG, we routinely purify IgG from sera because it is a simple one step procedure that results in concentrated antibody that produces a low background straining in the SRID, as well as in other assays such as ELISAs. In the five antibody preparations described in this study, all using three rabbits per project, we obtained approximately 250 mL at a final IgG concentration of ~10 mg/mL. As the optimal IgG antibody concentration used in the SRID was between 5 and 12 μL/mL, compared with 26 to 35 μL of the standard Reference Antiserum, the five antibodies prepared in rabbits were equivalent to 500 to greater than 1000 mL of the comparable Reference Antiserum prepared in sheep. Such yields are more than sufficient for preparing libraries of antibody reagents for pandemic preparedness and for use in evaluating pilot lots of pre‐pandemic vaccines. Future work will evaluate how well the rHA produced in the Expi293 expression system will perform when used as immunogens in a larger animal species such as sheep when much larger quantities of antibody are needed. It will also be interesting to explore whether antibody reagents produced in this manner will be applicable for determining potency of vaccines manufactured in other platforms.

In summary, we have described a simple mammalian cell transfection method to produce and purify recombinant influenza HA that can be used to generate HA antibody suitable for use in the influenza vaccine SRID potency assay. While the methodology has so far only been used to produce rHA for two H5 and one H2 hemagglutinin, the results suggest that this may be a workable alternative for reagent preparation, particularly in an emergency situation. The results also suggest a practical means by which an extensive library of pandemic reagents can easily be prepared in advance of such an emergency.

## 4 Materials and Methods

### 4.1 Cells and Viruses

Expi293F cells (Thermo Fisher Scientific) were maintained in suspension culture (orbital shaker at 125 RPM, 19 mm orbit) at 37°C, 80% Humidity, and 8% CO₂ in Expi293 Expression Medium (Thermo Fisher Scientific) for up to 30 passages.

### 4.2 Influenza HA Expression Plasmids

DNA expression plasmids were constructed as previously described [3]. Briefly, DNA sequences encoding the full‐length or ectodomain of the mature H5 HA were optimized for mammalian cell expression, synthesized, and cloned downstream of a sequence encoding a synthetic signal peptide (GenScript, Piscataway NJ).

### 4.3 Transfection and Purification of Influenza HA

Expi293F cells (3 x 10^6^/mL) were transfected using the ExpiFectamine 293 Transfection Kit (Thermo Fisher Scientific) per conditions outlined in the Expi293 Expression System User Guide (Thermo Fisher Scientific). *Vibrio cholerae* neuraminidase (Sigma‐Aldrich, Product No. N6514) was also added 1‐day post‐transfection to cultures of Ectodomain constructs to improve yields, presumably by liberating the hemagglutinin protein into the supernatant. Cultures were harvested 4 days post transfection.

Ectodomain HA cultures were harvested by spinning the cultures at 2500× *g* for 10 min. Subsequent purification of the supernatant was accomplished by filtering the supernatant with a 0.45 μm cellulose acetate filter. An equal volume of equilibration buffer (50 mM sodium phosphate pH 8.0, 300 mM NaCl, 25 mM imidazole) was then added to the supernatant, as well as 1 mL of PureCube 100 INDIGO Ni‐Agarose (Cube Biotech, Order No. 75110) resin per 100 mL of culture volume at the time of transfection. The supernatant/equilibration buffer/resin mixture was then rotated at 4°C for at least 1 h or overnight to allow resin to bind to the His‐tag.

After allowing time for binding, the mixture was spun at 700× *g* for 5 min. to separate out the resin. After pipetting off the supernatant, it was washed with 10–20 resin bed volumes of equilibration buffer. The spin/wash of the resin was repeated, and the resin was transferred to a gravity‐flow column with a stop‐cock in place. Once the resin settled, the column was washed once with 5 resin bed volumes of equilibration buffer before eluting with 5 bed volumes of elution buffer (50 mM sodium phosphate, 300 mM NaCl, 250 mM imidazole). Additional bed volumes of elution buffer were used as needed to check for more protein after the first 5 bed volumes. Optical densitometry was used to determine protein concentration. Eluted protein then underwent dialysis overnight into Size Exclusion Column (SEC) Buffer (50 mM Tris–HCl pH 8.0, 150 mM NaCl). The following day, the dialyzed protein was washed two times with additional SEC Buffer and concentrated using a 30 K regenerated cellulose membrane centrifugal filter unit (Amicon Ultra [Merck Millipore]) prior to SEC.

Full Length HA cultures were harvested by spinning the culture at 2500× *g* for 10 min. After discarding the supernatant, subsequent lysis of the cell pellets was accomplished by adding Extraction Buffer (100 mM Tris–HCl pH 8.0, 150 mM NaCl, 1 mM EDTA, containing 1% [w/v] n‐Dodecyl‐β‐D‐Maltopyranoside (DDM ‐ Anatrace Products LLC, Maumee, OH) and protease inhibitors (SigmaFAST Protease Inhibitor Cocktail tablet [Millipore Sigma] per 100 mL), followed by vortexing until no clumps remained; 10 mL of Extraction Buffer was used per 50 mL of initial culture volume. After incubating lysates on ice for 1 h, the lysate supernatant was collected by spinning out the cell debris twice at 23,000× *g* for 10 min. Following collection, the composition of lysate supernatant was adjusted to match Lentil Lectin Column Binding Buffer (0.5% [w/v] DDM, 20 mM Tris–HCl pH 7.4, 500 mM NaCl, 0.1 mM MnCl₂, 0.1 mM CaCl₂). Lentil Lectin Sepharose 4B resin (Cytiva) was then added to lysate supernatant (1.25–2.5 mL resin per 100 mL of initial transfection culture volume). The lysate supernatant/resin mixture was incubated at 4°C for at least 3 h or overnight to allow glycoproteins to bind to the resin.

After binding, the mixture was transferred to a gravity‐flow column and allowed to settle before the lysate supernatant was flowed through the resin. The settled resin was then washed with 10 resin bed volumes of Column Wash Buffer (0.25% [w/v] DDM, 20 mM Tris–HCl pH 7.4, 500 mM NaCl, 0.1 mM MnCl₂, 0.1 mM CaCl₂) before adding 5 resin bed volumes of Lentil Lectin Elution Buffer (0.02% [w/v] DDM, 500 mM methyl‐α‐D‐mannopyranoside (Sigma Aldrich)) and incubating 1 h before elution. Additional bed volumes of elution buffer were used as needed to check for more protein after the first 5 bed volumes. Optical densitometry was used to determine protein concentration. Eluted protein then underwent dialysis overnight into SEC buffer with 0.02% DDM. The dialyzed protein was washed with additional 0.02% DDM SEC Buffer and concentrated with a regenerated cellulose centrifugal concentrator unit.

Both ectodomain and full‐length antigens were further purified by size exclusion chromatography on a HiLoad Superdex 200 pg (Cytiva) column in SEC or DDM SEC buffer, respectively. Column fractions were evaluated for protein content and biological activity in HA assay before pooling and final concentration and determination of protein concentration.

### 4.4 Antibody Preparation

Purified protein was used to immunize New Zealand White rabbits using a standard immunization protocol under contract with SouthernBiotech, Birmingham, AL. All immunization projects were performed at SouthernBiotech's USDA registered Class R ‐ Research Facility (Certificate No. 64‐R‐0018) following SouthernBiotech's Custom Rabbit Animal Proposal Form No. FP‐0110, which is approved by Southern Biotech's IACUC. IgG was purified by Protein A affinity chromatography. Yields of the IgG antibodies used in this study: GY‐ED 261 mls @ 11.1 mg/mL; GY‐FL 259 mls @ 10.46 mg/mL; AS‐ED 265 mls @ 9.28 mg/mL; AS‐FL 255 mls @10.64 mg/mL; chicken‐Ohio ED 152 mls @ 12.8 mg/mL.

### 4.5 Western Blot and SDS‐PAGE Protein Analysis

Proteins in supernatants and cell lysates were resolved under reducing and non‐reducing conditions by SDS‐PAGE on 10‐well 4%–12% NUPAGE Bolt gels (Thermo Fisher Scientific) essentially as described previously [19]. Total proteins were visualized by Coomassie staining or transferred to a nitrocellulose membrane using an iBlot2 apparatus (Thermo Fisher Scientific), incubated with the primary antibody (mouse polyclonal antibody prepared by immunization with H5 A/gyrfalcon/Washington/41088‐6/2014 Reference Antigen) and followed by the horseradish peroxidase labeled sheep anti‐mouse IgG secondary antibody for detection (GE Healthcare). Images were captured using a G:Box iChemi XT imaging system (Syngene).

### 4.6 Hemagglutination and Hemagglutination Inhibition Assay

The hemagglutination and hemagglutination inhibition assays were performed in 96‐well plates (U‐bottom) by standard methods, essentially as described previously [20] using 0.5% chicken red blood cells suspended in PBS (pH 7.2).

### 4.7 Single‐Radial Immunodiffusion (SRID) Assay

The SRID assay was performed essentially as described previously [21, 22] and is based on the diffusion of detergent‐disrupted virus (or virus antigen) into an agarose gel containing specific influenza HA antibodies. Vaccine potency was determined by the parallel line bioassay method using reference and test vaccine dose–response curves (log antigen dilution versus log zone diameter). Replicates were included in each SRID assay, and assays were repeated on different days. The following WHO Reference Antigens and Antisera were used in the SRID analyses and comparisons:

1. CBER H5‐Ag‐1511 Reference Antigen is a BPL inactivated, whole virus preparation made from H5N8 A/gyrfalcon/Washington/41088–6/2014 grown in Madin Darby Canine Kidney (MDCK) cells.
2. CBER H5‐Ag‐1514 Reference Antigen is a zonally purified, BPL inactivated, whole virus preparation made from H5N8 A/gyrfalcon/Washington/41088–6/2014 grown in embryonated eggs.
3. CBER H5‐Ag‐2213 Reference Antigen is a lyophilized preparation of purified BPL inactivated whole virus made from H5N8 A/Astrakhan/3212/2020 grown in MDCK cells.
4. CBER H5‐Ag‐2301 Reference Antigen is a lyophilized preparation of purified formalin inactivated whole virus, made from H5N8 A/Astrakhan/3212/2020 grown in eggs.
5. CBER H2‐Ag‐2212 Reference Antigen is a lyophilized preparation of purified BPL inactivated whole virus made from H2N3 A/chicken/Ohio/494832/2007 grown in MDCK cells.
6. CBER H5‐Ab‐1515 antiserum reagent was prepared by immunization of sheep with bromelain cleaved HA from egg grown A/gyrfalcon/Washington/41088‐6/2014.
7. CBER H5‐Ab2313 antiserum reagent was prepared by immunization of sheep with bromelain cleaved HA from egg grown A/Astrakhan/3212/2020.
8. NIBSC 00/440 H2 antiserum reagent was prepared by immunization of sheep with bromelain cleaved HA from purified A/Japan/305/57.

## Author Contributions

**Marcus Odin:** investigation, writing – original draft, methodology. **Falko Schmeisser:** investigation, writing – review and editing. **Jackeline Soto:** investigation, writing – review and editing. **Jerry P. Weir:** conceptualization, investigation, writing – original draft, writing – review and editing.

## Conflicts of Interest

The authors declare no conflicts of interest.

## Supporting information


**Figure S1.** Size exclusion chromatography profiles of affinity purified A/gyrfalcon/Washington ectodomain (top) or full‐length rHA (bottom). HA values for individual fractions of the full‐length SEC are shown in the red bars.
**Figure S2.** Characterization of Expi293 expressed H2 rHA. Recombinant A/chicken/Ohio ectodomain HA from transfected Expi293 cells was purified and characterized by SDS‐PAGE under reducing conditions, stained with Coomassie blue (top left) or transferred to nitrocellulose membranes for analysis by Western blot (bottom left). rHA, pre‐SEC was analyzed for HA activity using chicken red blood cells and a starting protein concentration of 0.79 mg/mL in the 1:2 well (right).

## Data Availability

The authors declare that all relevant data supporting the findings of this study are available within the paper and its supplementary information files. Reagents generated in the study are available upon request.
