# Supplementary material for: A Rapid Virus‐Free Method for Producing Influenza HA Immunogen Needed for Preparation of Influenza Vaccine Potency Antisera Reagents
Source: Influenza Other Respir Viruses. 2024 Oct 23;18(10):e70024. doi: 10.1111/irv.70024 (PMC11497102; doi:10.1111/irv.70024)

Supplementary Figure 1 - Size exclusion chromatography profiles of affinity purified A/gyrfalcon/Washington ectodomain (top) or full-length rHA (bottom). HA values for individual fractions of the full-length SEC are shown in the red bars.

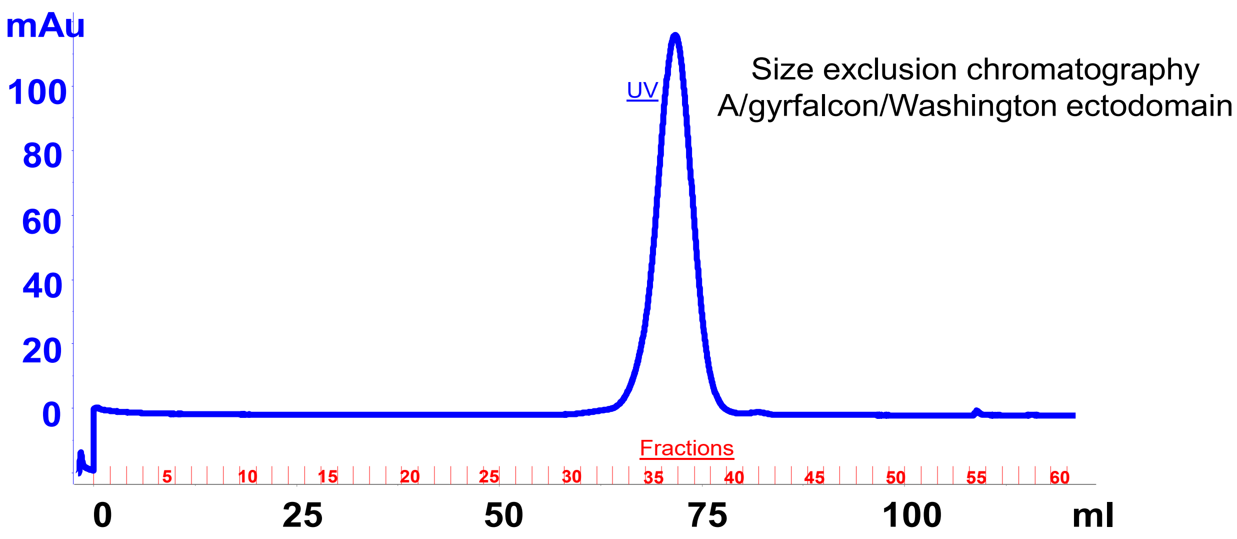

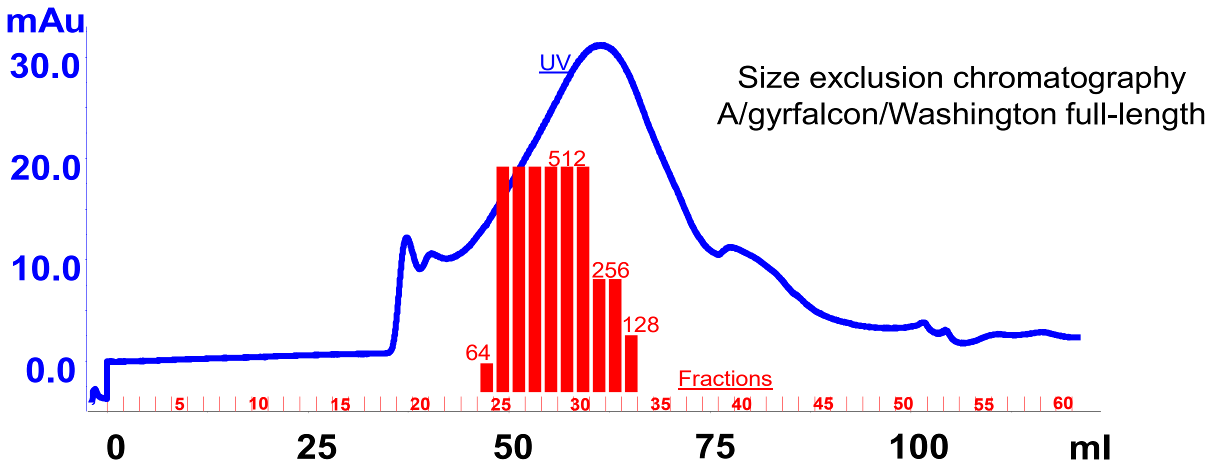

Supplementary Figure 2 - Characterization of Expi293 expressed H2 rHA. Recombinant A/chicken/Ohio ectodomain HA from transfected Expi293 cells was purified and characterized by SDS-PAGE under reducing conditions, stained with Coomassie blue (top left) or transferred to nitrocellulose membranes for analysis by Western blot (bottom left). rHA, pre-SEC was analyzed for HA activity using chicken red blood cells and a starting protein concentration of 0.79 mg/mL in the 1:2 well (right).

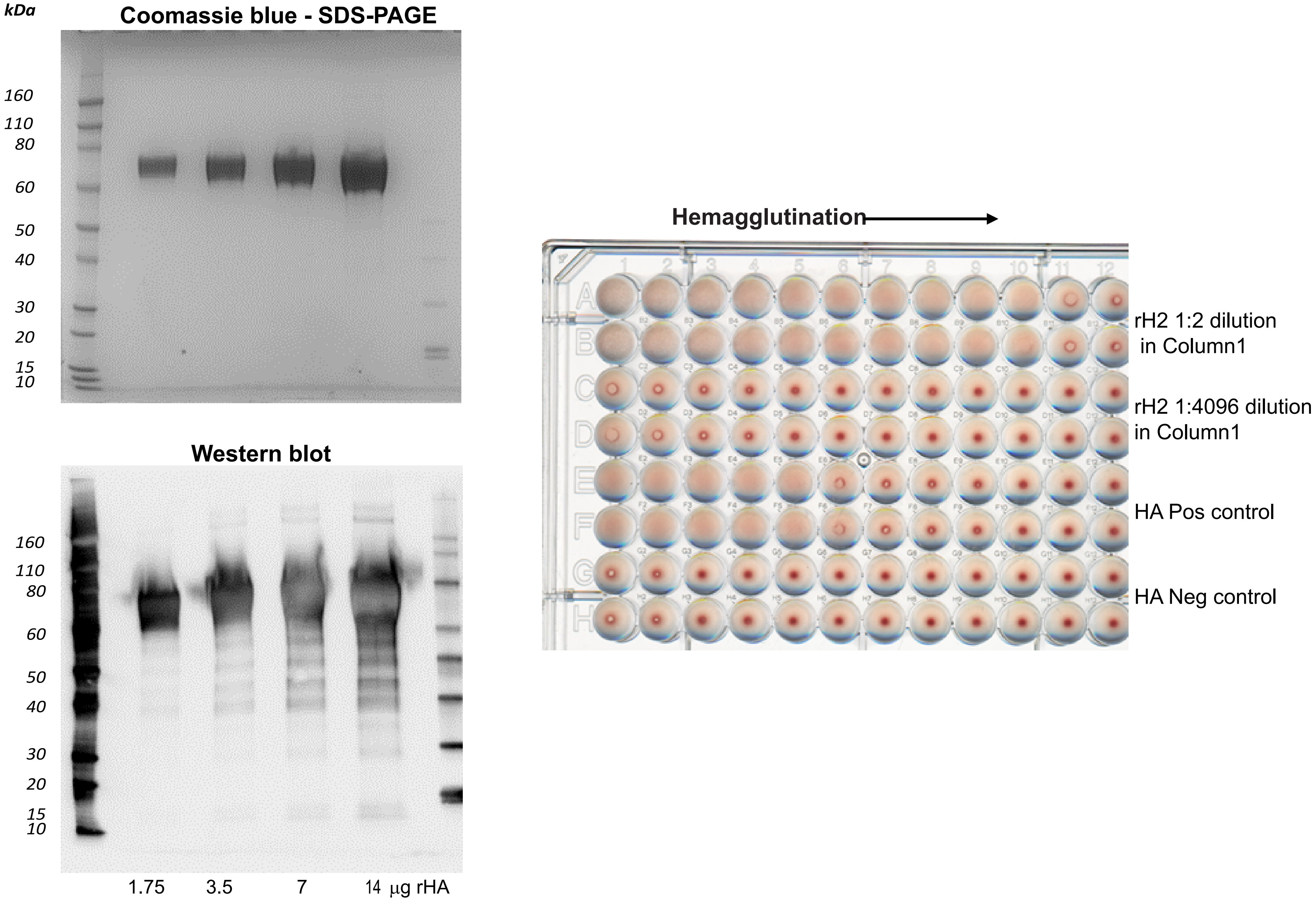

Supplement: Supplementary file 1 — Figure S1. Size exclusion chromatography profiles of affinity purified A/gyrfalcon/Washington ectodomain (top) or full‐length rHA (bottom). HA values for individual fractions of the full‐length SEC are shown in the red bars. Figure S2. Characterization of Expi293 expressed H2 rHA. Recombinant A/chicken/Ohio ectodomain HA from transfected Expi293 cells was purified and characterized by SDS‐PAGE under reducing conditions, stained with Coomassie blue (top left) or transferred to nitrocellulose membranes for analysis by Western blot (bottom left). rHA, pre‐SEC was analyzed for HA activity using chicken red blood cells and a starting protein concentration of 0.79 mg/mL in the 1:2 well (right). [file IRV-18-e70024-s001.pdf]
